# Author Correction: A genomic catalogue of soil microbiomes boosts mining of biodiversity and genetic resources

**DOI:** 10.1038/s41467-023-44072-7

**Published:** 2023-12-06

**Authors:** Bin Ma, Caiyu Lu, Yiling Wang, Jingwen Yu, Kankan Zhao, Ran Xue, Hao Ren, Xiaofei Lv, Ronghui Pan, Jiabao Zhang, Yongguan Zhu, Jianming Xu

**Affiliations:** 1 Institute of Soil and Water Resources and Environmental Science, College of Environmental and Resource Sciences, Zhejiang University, Hangzhou, 310058 China; 2 Zhejiang Provincial Key Laboratory of Agricultural Resources and Environment, Zhejiang University, Hangzhou, 310058 China; 3 ZJU-Hangzhou Global Scientific and Technological Innovation Center, Hangzhou, 311200 China; 4 Department of Environmental Engineering, China Jiliang University, Hangzhou, 310018 China; 5 State Key Laboratory of Soil and Sustainable Agriculture, Institute of Soil Science, Chinese Academy of Sciences, Nanjing, 210008 China; 6 Research Center for Eco-environmental Sciences, Chinese Academy of Sciences, Beijing, 100085 China

**Keywords:** Metagenomics, Soil microbiology

Correction to: *Nature Communications* 10.1038/s41467-023-43000-z, published online 11 November 2023

The original version of this Article contained an error in the Abstract. The correct version states “untapped reservoir” in place of “untapped reservo)ir”. The original version of this Article also contained an error in the Methods section. The correct version states “SNV, Pangenome analysis of SMAG” in place of “SNV, Pangenome analysis of SAMG”. This has been corrected in both the PDF and HTML versions of the Article.

