# Supplementary material for: A genomic catalogue of soil microbiomes boosts mining of biodiversity and genetic resources
Source: Nat Commun. 2023 Nov 11;14:7318. doi: 10.1038/s41467-023-43000-z (PMC10640626; doi:10.1038/s41467-023-43000-z)
Supplement: Supplementary file 1 — Supplementary information [file 41467_2023_43000_MOESM1_ESM.pdf]

A Genomic Catalogue of Soil Microbiomes Boosts Mining of Biodiversity and Genetic Resources

Supplementary Figures legends

Supplementary Fig. 1. Overview of the genome quality from SMAG. a, The number of reconstructed MAGs at different sequencing depths (Number of clean reads). **b,** The number of reconstructed MAGs per metagenome. **c,** The completeness and genome sizes for the SGBs. **d,** The contamination and genome sizes for the SGBs. **e,** Completeness and contamination scores for the SGBs. **(c, d and e)** are all colored by their quality classification category. **f,** Distribution of the level of strain heterogeneity estimated for the species-level MAGs. from CheckM. Dashed vertical lines are the median strain heterogeneity of medium and high-quality MAGs, respectively.

Supplementary Fig. 2. Overview of the reconstructed SGBs and taxonomic assignment. a, The distribution of the number of MAGs across kSGBs and uSGBs. **b,** The relationship between SGBs and corresponding reference genomes. **c,** The genome size of kSGBs and uSGBs across phyla (n = 21,077). **d,** Identification of relative evolutionary divergence (RED) cutoffs for phylogenetic clustering in the SMAG tree.

Supplementary Fig. 3. Functional and intraspecies genomic annotations. a, The comparison of the number of KEGG pathways between kSGBs and uSGBs. **b, The proportion of each COG functional category between kSGBs and uSGBs. c,** The length of 107 pangenomes and the number of genomes in each pangenome. Dash horizontal line is the average pangenome length. **d,** Correlations between the percentage of core genes and the number of genomes. **e,** Correlations between the percentage of core genes and the size of the genomes. **f,** The density of SNVs for kSGBs and uSGBs across the dominant phyla.

Supplementary Fig. 4. Histogram of BGC sizes and the representative length BGCs. a, Distribution of BGC length. The SMAG catalogue contains complex types of multigene BGCs, most of which were fragmented BGCs. **b-f,** Five NRPS clusters with a length >100kb.

Supplementary Fig. 5. Overview of the CRISPR and Cas protein identified from the SMAG catalogue. a, The number of spacers identified across the reconstructed MAGs. **b,** The comparison of identified spacers between uSGBs and kSGBs (n = 662 MAGs). **c,** The number of Cas proteins identified across the reconstructed MAGs (n = 563 MAGs). **d,** The Cas proteins were identified as novel and old Cas across Cas protein types.

Supplementary Fig. 6. Quality of virus and virus-host gene ratio. a, The quality of the viruses identified across dominant phyla. b, The distribution of the viral length. e, The overview of virus and host gene ratio was identified from the SMAG.

Supplementary Figures

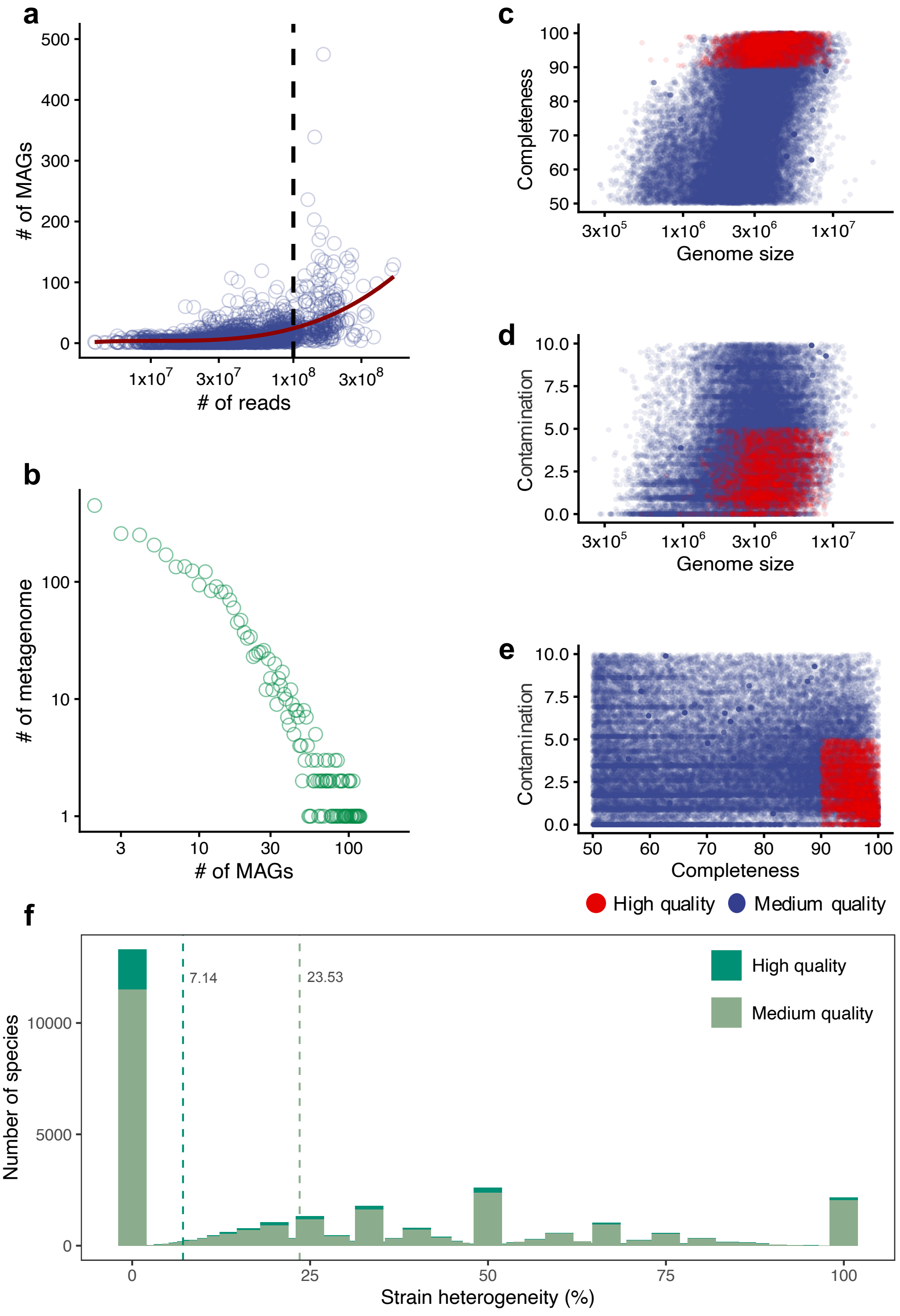

Supplementary Fig. 1: Overview of the genome quality from SMAG. a, The number of reconstructed MAGs at different sequencing depths (Number of clean reads). **b,** The number of reconstructed MAGs per metagenome. **c,** The completeness and genome sizes for the SGBs. **d,** The contamination and genome sizes for the SGBs. **e,** Completeness and contamination scores for the SGBs. **(c, d and e)** are all colored by their quality classification category. **f,** Distribution of the level of strain heterogeneity estimated for the species-level MAGs. from CheckM. Dashed vertical lines are the median strain heterogeneity of medium and high-quality MAGs, respectively.

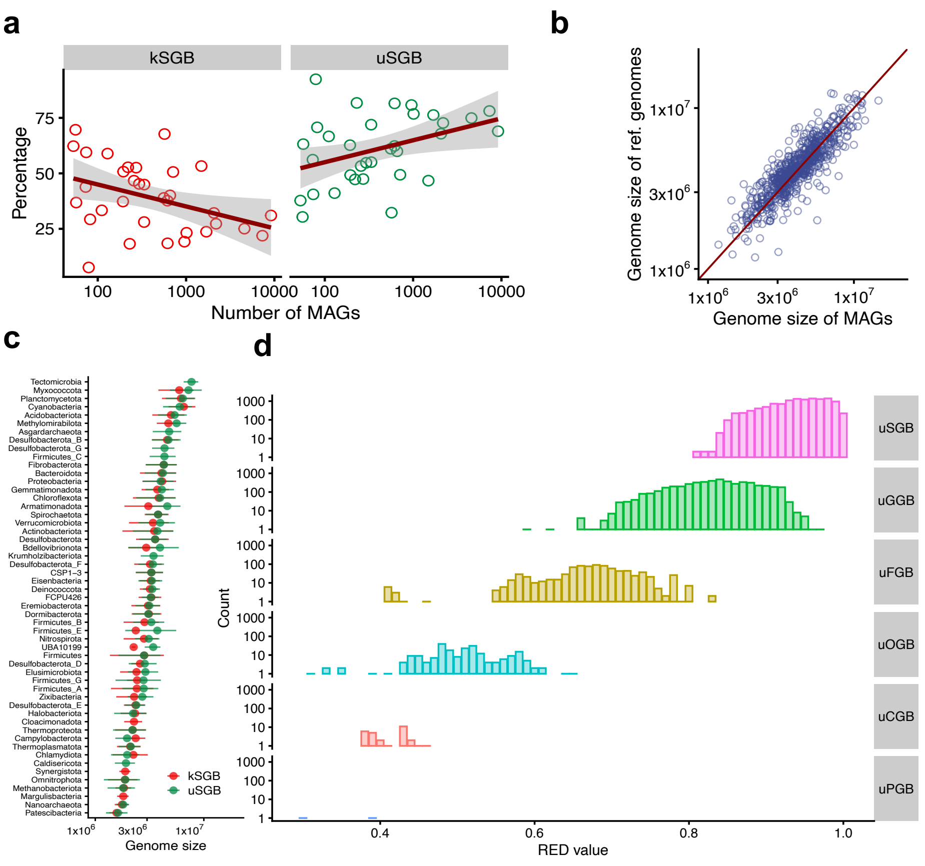

Supplementary Fig. 2: Overview of the reconstructed SGBs and taxonomic assignment. **a,** The distribution of the number of MAGs across kSGBs and uSGBs. **b,** The relationship between SGBs and corresponding reference genomes. **c,** The genome size of kSGBs and uSGBs across phyla (n= 21,077). **d,** Identification of relative evolutionary divergence (RED) cutoffs for phylogenetic clustering in the SMAG tree.

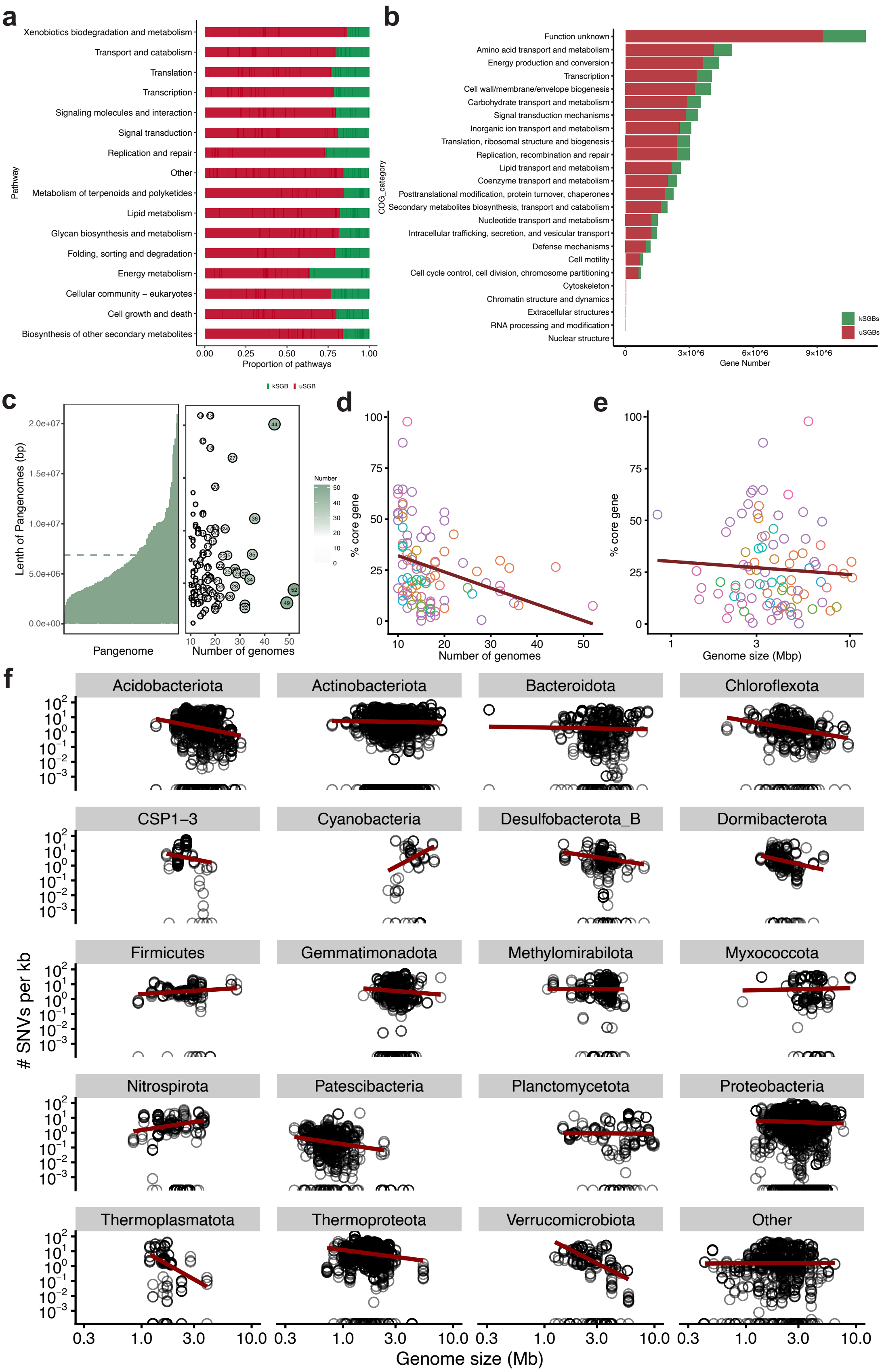

Supplementary Fig. 3: Functional and intraspecies genomic annotations. a, The comparison of the number of KEGG pathways between kSGBs and uSGBs. **b, The proportion**

of each COG functional category between kSGBs and uSGBs. c, The length of 107 pangenomes and the number of genomes in each pangenome. Dash horizontal line is the average pangenome length. **d,** Correlations between the percentage of core genes and the number of genomes. **e,** Correlations between the percentage of core genes and the size of the genomes. **f,** The density of SNVs for kSGBs and uSGBs across the dominant phyla.

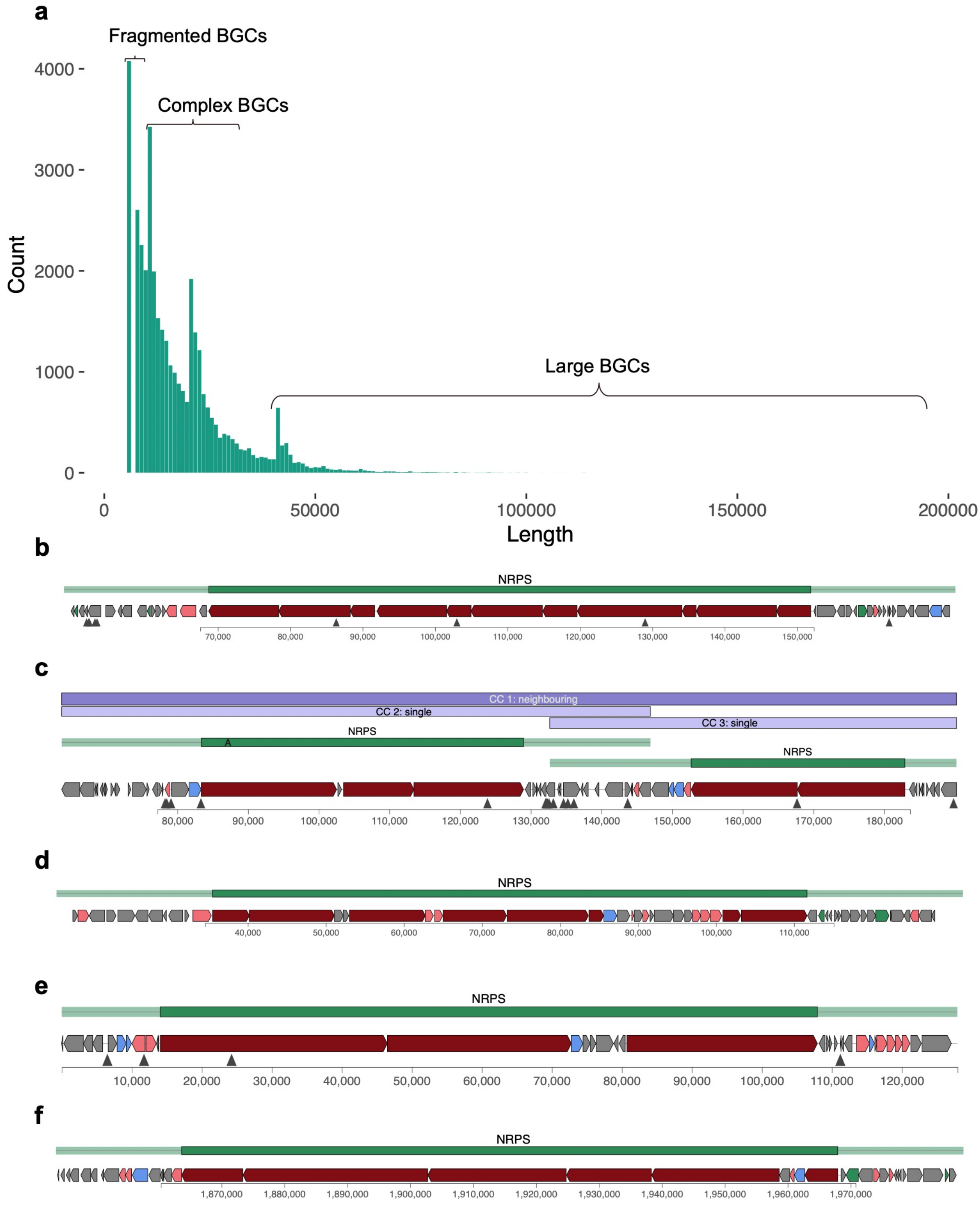

Supplementary Fig. 4: Histogram of BGC sizes and the representative length BGCs. a, Distribution of BGC length. The SMAG catalogue contains complex types of multigene BGCs, most of which were fragmented BGCs. **b-f,** Five NRPS clusters with a length >100kb.

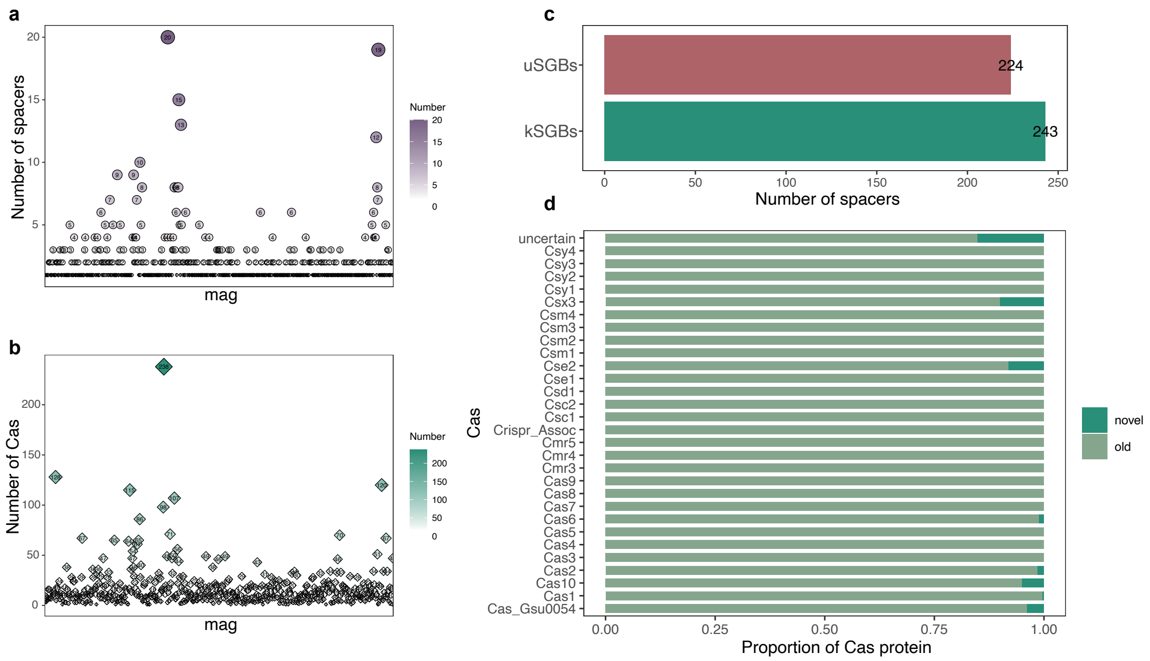

Supplementary Fig. 5: Overview of the CRISPR and Cas proteins identified from the SMAG catalogue. a, The number of spacers identified across the reconstructed MAGs (n = 662 MAGs). **b,** The comparison of identified spacers between uSGBs and kSGBs (n = 563 MAGs). **c,** The number of Cas proteins identified across the reconstructed MAGs. **d,** The Cas proteins were identified as novel and old Cas across Cas protein types.

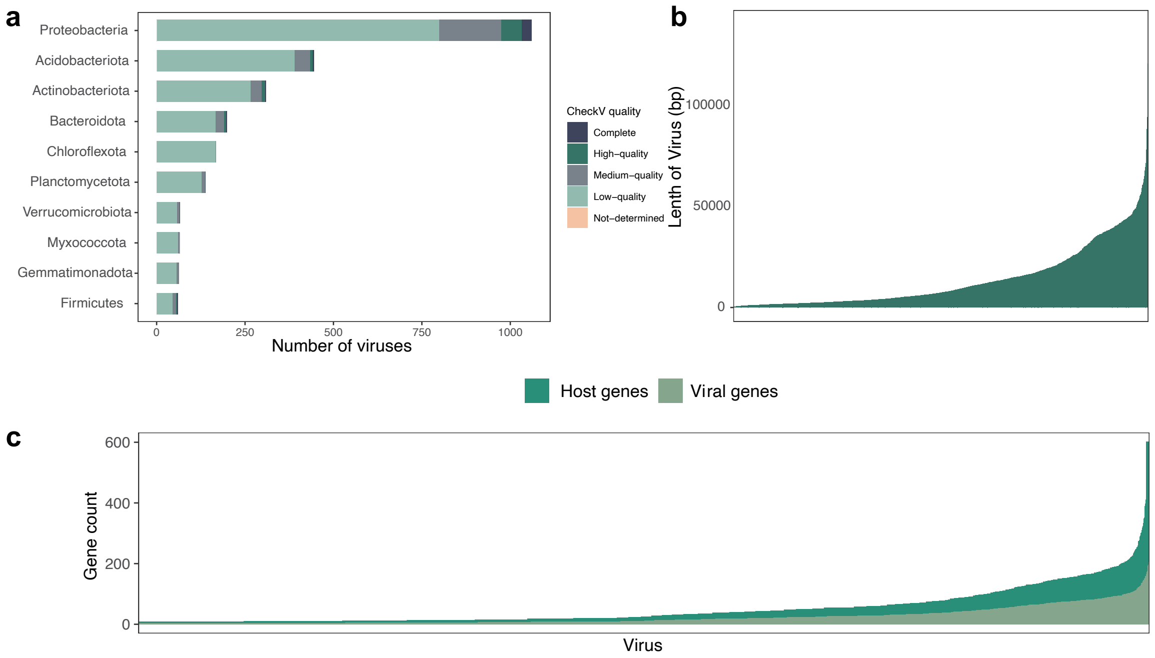

Supplementary Fig. 6: Quality of virus and virus-host gene ratio. a, The quality of the viruses identified across dominant phyla. **b,** The distribution of the viral length. **e,** The overview of virus and host gene ratio was identified from the SMAG.
