# Supplementary material for: A genomic catalogue of soil microbiomes boosts mining of biodiversity and genetic resources
Source: Nat Commun. 2023 Nov 11;14:7318. doi: 10.1038/s41467-023-43000-z (PMC10640626; doi:10.1038/s41467-023-43000-z)
Supplement: Supplementary file 3 — Description of Additional Supplementary Files [file 41467_2023_43000_MOESM3_ESM.docx]

# Description of Additional Supplementary Files

Supplementary Data 1: Detailed information about the soil metagenomes used to reconstruct metagenome-assembled genomes (MAGs).

Supplementary Data 2: The quality and taxonomy information of the 40,039 reconstructed MAGs and representative 21,077 SGBs; Summary of the studied CPR and DPANN MAGs in the present study.

Supplementary Data 3: The profiles of MAGs used to generate pangenome and single nucleotide variants (SNVs) catalogue.

Supplementary Data 4: The length and classification profiles of biosynthetic gene clusters (BGCs)(>5kb) identified from the SMAG catalogue; core biosynthetic gene sequence identity matrix and KO assignment of the two largest BGCs from SMAG and GEM.

Supplementary Data 5: The profiles of MAGs used to identify spacers and cas proteins; The information of cas proteins.

Supplementary Data 6: The profiles of MAGs and predict virus-host associations and the profiles of viruses identified from the SMAG catalogue.
