# Supplementary material for: A genomic catalogue of soil microbiomes boosts mining of biodiversity and genetic resources
Source: Nat Commun. 2023 Nov 11;14:7318. doi: 10.1038/s41467-023-43000-z (PMC10640626; doi:10.1038/s41467-023-43000-z)
Supplement: Supplementary file 10 — Reporting Summary [file 41467_2023_43000_MOESM10_ESM.pdf]

nature portfolio

Corresponding author(s): Jianming Xu

Last updated by author(s): Oct. 14, 2023

# Reporting Summary

Nature Portfolio wishes to improve the reproducibility of the work that we publish. This form provides structure for consistency and transparency in reporting. For further information on Nature Portfolio policies, see our Editorial Policies and the Editorial Policy Checklist.

## Statistics

For all statistical analyses, confirm that the following items are present in the figure legend, table legend, main text, or Methods section.

| n/a | Confirmed | |
|---|---|---|
| ☐ | ☒ | The exact sample size (*n*) for each experimental group/condition, given as a discrete number and unit of measurement |
| ☒ | ☐ | A statement on whether measurements were taken from distinct samples or whether the same sample was measured repeatedly |
| ☐ | ☒ | The statistical test(s) used AND whether they are one- or two-sided<br>*Only common tests should be described solely by name; describe more complex techniques in the Methods section.* |
| ☒ | ☐ | A description of all covariates tested |
| ☒ | ☐ | A description of any assumptions or corrections, such as tests of normality and adjustment for multiple comparisons |
| ☐ | ☒ | A full description of the statistical parameters including central tendency (e.g. means) or other basic estimates (e.g. regression coefficient) AND variation (e.g. standard deviation) or associated estimates of uncertainty (e.g. confidence intervals) |
| ☐ | ☒ | For null hypothesis testing, the test statistic (e.g. *F*, *t*, *r*) with confidence intervals, effect sizes, degrees of freedom and *P* value noted<br>*Give P values as exact values whenever suitable.* |
| ☒ | ☐ | For Bayesian analysis, information on the choice of priors and Markov chain Monte Carlo settings |
| ☒ | ☐ | For hierarchical and complex designs, identification of the appropriate level for tests and full reporting of outcomes |
| ☒ | ☐ | Estimates of effect sizes (e.g. Cohen's *d*, Pearson's *r*), indicating how they were calculated |

*Our web collection on statistics for biologists contains articles on many of the points above.*

## Software and code

Policy information about availability of computer code

| | |
|---|---|
| Data collection | fastq-dump (v2.9.6) https://ftp-trace.ncbi.nlm.nih.gov/sra/sdk<br>Trimmomatic (v2.39) https://github.com/usadellab/Trimmomatic<br>MEGAHIT (v1.2.9) https://github.com/voutcn/megahit<br>Metabat (v2.12.1) https://bitbucket.org/berkeleylab/metabat<br>MaxBin (v2.2.6) https://sourceforge.net/projects/maxbin/<br>CONCOCT https://github.com/BinPro/CONCOCT<br>metaWRAP(v1.2.1) https://github.com/bxlab/metaWRAP<br>CheckM (v1.0.11) https://github.com/Ecogenomics/CheckM<br>Barrnap (v.0.9) https://github.com/tseemann/barrnap<br>tRNAscan-SE (v.2.0.9) https://github.com/UCSC-LoweLab/tRNAscan-SE<br>dRep (v2.2.4) https://github.com/MrOlm/drep<br>Mash (v2.3) https://github.com/marbl/mash<br>Mummer (v4.0.0) https://github.com/gmarcais/mummer<br>FastANI https://github.com/ParBLiSS/FastANI<br>GTDB-TK (v.1.6.0) https://github.com/Ecogenomics/GtdbTk<br>PhyloPhlAn (v3.0.60) https://github.com/biobakery/phylophlan<br>Diamond (v0.9.14.115) https://github.com/bbuchfink/diamond<br>mafft (version v7.310) https://github.com/The-Bioinformatics-Group/Albiorix/wiki/mafft<br>trimal (version 1.4rev15) https://github.com/scapella/trimal |

RAxML (version 8.1.12) https://github.com/stamatak/standard-RAxML
FastTree (version 2.1.10) https://github.com/PavelTorgashov/FastTree
HMM https://github.com/guyz/HMM
Prodigal (v2.6.3) https://github.com/hyattpd/prodigal/wiki
BWA (v0.7.17) https://github.com/lh3/bwa
Samtools (v1.10) https://github.com/samtools/
IQ-TREE (v1.6.6) http://www.cibiv.at/software/iqtree
antiSMASH(v6.1) https://github.com/antismash/antismash
BiG-SCAPE https://github.com/medema-group/BiG-SCAPE
Clustal (v2.1) http://www.clustal.org/
BlastKOALA (v.2.21) https://www.kegg.jp/blastkoala/
Roary (v3.12.0) https://github.com/sanger-pathogens/Roary
MMseqs2 https://github.com/soedinglab/MMseqs2
eggNOG-mapper (v2.1.6) https://github.com/eggnogdb/eggnog-mapper
PILER-CR https://github.com/widdowquinn/pilercrpy
VirSorter2 (v2.0 alpha) https://github.com/jiarong/VirSorter2
CheckV (v1.0) https://bitbucket.org/berkeleylab/CheckV

Data analysis

The workflow used to generate the genome, taxonomic analysis, and functional annotation, alongside the BGCs, pan-genome, SNV annotations, and virus predictions and scripts used to generate the figures are described at GitHub repository through https://github.com/Caiyulu-818/SMAG/releases/tag/v1.0. All statistical analyses for generating figures were performed using the R environment v4.1.2

For manuscripts utilizing custom algorithms or software that are central to the research but not yet described in published literature, software must be made available to editors and reviewers. We strongly encourage code deposition in a community repository (e.g. GitHub). See the Nature Portfolio guidelines for submitting code & software for further information.

## Data

Policy information about availability of data

All manuscripts must include a data availability statement. This statement should provide the following information, where applicable:
- Accession codes, unique identifiers, or web links for publicly available datasets
- A description of any restrictions on data availability
- For clinical datasets or third party data, please ensure that the statement adheres to our policy

The raw sequence data of the in-house samples reported in this paper and the 16,530 uSGBs of the SMAG catalogue have been deposited to NCBI SRA and GenBank under the bioproject accession number: PRJNA983538. For the bulk download, all the MAGs, SNV catalogues and viruses predicted the SMAG has been deposited in Zenodo repository through https://doi.org/10.5281/zenodo.7341719 (ref 100) and also be available in the freely accessible interface-web of the SMAG catalogue (https://smag.microbmalab.cn). The source data underlying Figs. 1–6 and Supplementary Figs. 1-6 are provided as Source Data files and have been deposited in the Figshare database (https://doi.org/10.6084/m9.figshare.23298791). The databases used in this study include GEM catalog (https://genome.jgi.doe.gov/portal/GEMs/GEMs.home.html), the UHGG (https://ftp.ebi.ac.uk/pub/databases/metagenomics/mgnify_genomes/), and GTDB database Release 202 (https://data.ace.uq.edu.au/public/gtdb/data/releases/release202/).

## Research involving human participants, their data, or biological material

Policy information about studies with human participants or human data. See also policy information about sex, gender (identity/presentation), and sexual orientation and race, ethnicity and racism.

| | |
|---|---|
| Reporting on sex and gender | NA |
| Reporting on race, ethnicity, or other socially relevant groupings | NA |
| Population characteristics | NA |
| Recruitment | NA |
| Ethics oversight | NA |

Note that full information on the approval of the study protocol must also be provided in the manuscript.

# Field-specific reporting

Please select the one below that is the best fit for your research. If you are not sure, read the appropriate sections before making your selection.

☐ Life sciences ☐ Behavioural & social sciences ☒ Ecological, evolutionary & environmental sciences

For a reference copy of the document with all sections, see nature.com/documents/nr-reporting-summary-flat.pdf

# Ecological, evolutionary & environmental sciences study design

All studies must disclose on these points even when the disclosure is negative.

| | |
|---|---|
| Study description | The study conduct the first large-scale excavation of soil microbial dark matter by constructing an informational public resource database and explore soil microbial dark matter from metagenomes by metagenomic analysis. |
| Research sample | We downloaded 2,941 soil metagenomes from the NCBI Sequence Read Archive (SRA) publicly available with file sizes exceeding 2GB from different countries which cover 9 soil ecosystems and about 363 in-house data were sampled. |
| Sampling strategy | In-house samples for this study were sourced from field sampling conducted using a uniform sampling protocol. Within this protocol, five soil cores were randomly taken within plots to a depth of up to 15 cm and combined into one composite sample to ensure the randomness and representativeness of the sample. All soil samples were kept cool using dry ice until visible roots and stones were removed. And then all clean soils were stored at -80°C until DNA extraction. In all cases, DNA extraction of 400 mg of soil in each sample was performed using MP FastDNA SPIN Kits 385 for soil (MP Biomedicals, Solon, OH, USA) according to the manufacturer's instructions and DNA was purified and concentrated using Qubit fluorometric quantitation (Thermo Fisher Scientific, 388 Waltham, MA, USA). Purified DNA was stored at -20°C for sequencing. Metagenomic sequencing from each soil sample was conducted by Illumina HiSeq 4000 or Illumina novaseq pe150 (Illumina, San Diego, CA, USA), generating 150 bp paired end reads. (see methods (sampling sequencing, in the manuscript) |
| Data collection | Soil metagenomes were downloaded using sratoolkit from the NCBI Sequence Read Archive (SRA) publicly available with file sizes exceeding 2GB in 2019-2020 by Caiyu Lu. |
| Timing and spatial scale | SRA soil metagenomic samples were collected at the global scale across 9 ecosystems from Europe, Asia, African and the north American by searching in the SRA of NCBI (No time limited and filtering). In-house samples original from China (348) and were largely collected between 2018 and 2019, and from Europe (15) were sampled in 2020. |
| Data exclusions | Small public datasets with an SRA file size <2Gb (shallow sampling depth) and without longitude and latitude (Insufficient sampling information) were excluded. |
| Reproducibility | Data were collected from published literatures and the measurements are described in the methods<br>All files and code required to repeat the experiments are provided in https://github.com/Caiyulu-818/SMAG/releases/tag/v1.0. We do not directly carry out any field measurement. |
| Randomization | Sample collection randomization within sites was determined by individual data sources prior to our study starting. Any conditions of the soil metagenomes (e.g. geographic location or biome type) were already determined before our study began. We do not directly carry out any field measurement. |
| Blinding | We do not directly carry out any field measurement. |

Did the study involve field work? ☐ Yes ☒ No

# Reporting for specific materials, systems and methods

We require information from authors about some types of materials, experimental systems and methods used in many studies. Here, indicate whether each material, system or method listed is relevant to your study. If you are not sure if a list item applies to your research, read the appropriate section before selecting a response.

## Materials & experimental systems

| n/a | Involved in the study |
|---|---|
| ☒ | ☐ Antibodies |
| ☒ | ☐ Eukaryotic cell lines |
| ☒ | ☐ Palaeontology and archaeology |
| ☒ | ☐ Animals and other organisms |
| ☒ | ☐ Clinical data |
| ☒ | ☐ Dual use research of concern |
| ☒ | ☐ Plants |

## Methods

| n/a | Involved in the study |
|---|---|
| ☒ | ☐ ChIP-seq |
| ☒ | ☐ Flow cytometry |
| ☒ | ☐ MRI-based neuroimaging |
